# The interplay between individual social behavior and clinical symptoms in small clustered groups

**DOI:** 10.1186/s12879-017-2623-2

**Published:** 2017-07-26

**Authors:** Piero Poletti, Roberto Visintainer, Bruno Lepri, Stefano Merler

**Affiliations:** 1 Department of Policy Analysis and public Management, Dondena Centre for Research on Social Dynamics and public Policy, Università Commerciale L. Bocconi, via Rontgen 1, Milan, Italy; 2 Bruno Kessler Foundation, via Sommarive 18, Trento, Italy

**Keywords:** Mixing patterns, Time-varying networks, Risk factor, Bluetooth proximity, Influenza-like illness

## Abstract

**Background:**

Mixing patterns of human populations play a crucial role in shaping the spreading paths of infectious diseases. The diffusion of mobile and wearable devices able to record close proximity interactions represents a great opportunity for gathering detailed data on social interactions and mixing patterns in human populations. The aim of this study is to investigate how social interactions are affected by the onset of symptomatic conditions and to what extent the heterogeneity in human behavior can reflect a different risk of infection.

**Methods:**

We study the relation between individuals’ social behavior and the onset of different symptoms, by making use of data collected in 2009 among students sharing a dormitory in a North America university campus. The dataset combines Bluetooth proximity records between study participants with self-reported daily records on their health state. Specifically, we investigate whether individuals’ social activity significantly changes during different symptomatic conditions, including those defining Influenza-like illness, and highlight to what extent possible heterogeneities in social behaviors among individuals with similar age and daily routines may be responsible for a different risk of infection for influenza.

**Results:**

Our results suggest that symptoms associated with Influenza-like illness can be responsible of a reduction of about 40% in the average duration of contacts and of 30% in the daily time spent in social interactions, possibly driven by the onset of fever. However, differences in the number of daily contacts were found to be not statistically significant. In addition, we found that individuals who experienced clinical influenza during the study period were characterized by a significantly higher social activity. In particular, both the number of person-to-person contacts and the time spent in social interactions emerged as significant risk factors for influenza infection.

**Conclusions:**

Our findings highlight that Influenza-like illness can remarkably reduce the social activity of individuals and strengthen the idea that the heterogeneity in social habits among individuals can significantly contribute in shaping differences among the individuals’ risk of infection.

**Electronic supplementary material:**

The online version of this article (doi:10.1186/s12879-017-2623-2) contains supplementary material, which is available to authorized users.

## Background

Mixing patterns of human populations play a crucial role in shaping the spreading paths of infectious diseases [1–3]. Technological advances and the diffusion of mobile and wearable devices, such as smartphones and radio-frequency identification (RFID) sensor systems, are enhancing our ability of gathering high-resolution data on proximity and face-to-face interactions [4–9]. Recently, the usage of wireless sensor network technologies and, more in general, of devices capable of sensing spatial proximity over different scales have been proposed to investigate how the community structure of contacts can affect the transmission dynamics of infectious diseases [10]. This type of data has also been used to simulate the potential spread of the infection within small but strongly connected communities, such as schools [6, 11], during a conference [12] and within hospitals [13–16].

Contact patterns in human populations are characterized by a high variability driven by working and schooling activities, individuals’ age and socio-demographic characteristics such as the household composition and the site of residence. Nonetheless, remarkable changes in individual social behavior may occur over time and social activity levels may be different among individuals characterized by similar age and daily routines. The proposed analysis focuses on a relatively small cluster of individuals representing a strongly connected community (e.g. hanging out and sleeping at the same places). The aim of this study is to investigate how social interactions are affected by the onset of symptomatic conditions and to what extent the heterogeneity in human behavior can reflect a different risk of infection. To do this, we make use of records about the presence of specific symptoms combined with proximity data collected between January and March 2009 among undergraduate students that share a dormitory in a campus of a major university in North America [17]. We define different measures of individuals’ social activity level and a dynamic contact network between study participants, based on Bluetooth proximity records. We perform a statistical analysis on whether or not the individuals’ time spent in social interactions, and the number and duration of daily contacts, significantly changes while experiencing different types of symptomatic conditions, including those defining Influenza-like illness. Finally, we investigate to what extent possible heterogeneities in individuals’ social activity level might be responsible of a significantly different risk of infection, providing some insights on features of social behavior that can be considered as risk factors for the onset of clinical influenza.

## Methods

### Proximity data and symptoms records

The proposed analysis is based on a dataset collected as part of a longitudinal study carried out between the 10th of January and the 10th of March 2009 including 74 undergraduate students uniformly distributed among four academic years and representing the 80% of the total residents of a dormitory at the campus of a major university in North America [17]. Study participants were asked to daily self report the presence of specific symptoms, and were equipped with a smartphone incorporating a mobile sensing software designed for recording physical proximity between involved individuals during the study period. The software scanned for Bluetooth wireless devices in proximity every 6 min, a compromise between sensing short-term social interactions and battery life [5]. During the same period of time, study participants daily reported whether or not they were experiencing the following symptoms: i) fever, ii) sore throat and cough, iii) runny nose, congestion or sneezing, iv) nausea, vomiting or diarrhea. The study had IRB approval by the Massachusetts Institute of Technology Committee on the Use of Humans as Experimental Subjects (MIT COUHES) and written informed consent from participants. More details are available in [17]. The datasets used in this article can be found in the Additional file 1: S1 and in [18].

### Individual social activity and dynamic proximity network

Connections between two individuals were defined as potential interactions between two study participants (e.g. verbal or physical contacts) that may have occurred as a consequence of their physical proximity. No cut-off values on the time spent in physical proximity were considered to define potential individuals’ interactions. A time-varying network between study participants was obtained by computing time dependent adjacency matrices *C*(*t*), in which each entry *C*_*j,i*_(*t*) defines the amount of time spent by individual *i* in physical proximity with individual *j* at each time step *t*. Connections reported in the study are undirected, therefore resulting adjacency matrices are symmetrical (i.e., *C*_*i,j*_ = *C*_*j,j*_ for any *t*). The intensity of social activity within the network considered was investigated by aggregating the time spent in proximity interactions between study participants within the same day. Specifically, the latter was computed as the product of the duration and counts of time slots in which physical proximity between individuals was recorded by the Bluetooth devices. Such aggregation has been shown to be a good approximation to analyze the temporal variation of a dynamic network characterizing interactions within a given population [12]. Proximity records defining the relative intensity of connections between study participants over time are therefore used to derive a set of proxy measures of the number and duration of contacts between individuals and of the overall time spent in social interactions. The network defined by proximity records associated with study participants is considered as a representative case study of the heterogeneity of social interactions occurring within a small but strongly connected cluster of individuals.

### The relationship between symptoms and human behavior

**Fig. 1:**
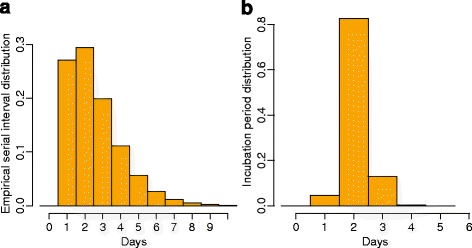
Probability density functions associated with the empirical serial interval (**a**) and the duration of the incubation period (**b**) of influenza, as estimated in [22]

Possible changes in human behavior triggered by symptomatic episodes were assessed by considering individuals who experienced a given symptom and by comparing their number and duration of social interactions during days in which the symptom was reported with respect to the other days. In order to highlight which are the features of social behavior that may influence the individual risk of infection we analyze the social activity level, characterizing individuals who reported symptoms compliant with an Influenza-like illness with respect to who did not. In this case, a Wilcoxon test was performed to assess the statistical significance (*p*-value <0.05) of differences detected across these two mutually exclusive groups of individuals. Finally, we investigate the potential transmission events occurred among study participants who could have experienced clinical influenza, by modeling contacts between individuals as driven by the dynamic network defined by Bluetooth proximity records. Possible epidemiological links were identified by considering the onset of symptoms associated with Influenza-like illness [19–21] and on the basis of estimates on the duration of the incubation period and the serial interval coming from the analysis of the 2009 H1N1 pandemic in UK (see Fig. 1) [22]. More details on this analysis can be found in the Additional file 2: S2.

## Results

### Individual social activity

**Fig. 2:**
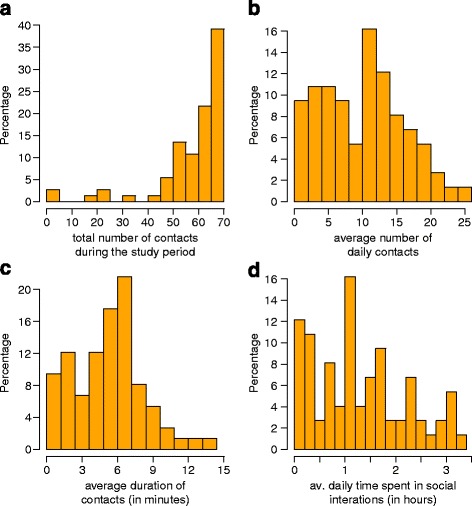
Distribution of **a** the total number of contacts during the study period, **b** the average number of contacts per day, **c** the average duration of contacts, **d** the average time spent in social interaction per day, according to physical proximity records collected during the study period

The network of contacts defined by all connections between the study participants recorded from January 10th to March 10th can be classified as a connected mesh, where all pairs of nodes x, y in the network are connected through a path defined by social interactions between study participants that leads from x to y. Each study participant was found at least once in physical proximity with at least one other study participant. Furthermore, about 90% of study participants recorded contacts with more than 40 individuals, corresponding to more than 50% of the considered individuals (see Fig. 2a). However, when the dynamic proximity network is considered, it emerges that, on average, a study participant had contact with 10 other individuals per day (sd 6.12, see Fig. 2b), spending on average 5.18 min per contact per day (sd 2.98 see Fig. 2c) and 1.29 h per day in social interactions with individuals sleeping at the dormitory (sd 0.91 see Fig. 2d). The daily number of contacts resulted strongly correlated with both the daily time spent in social interactions (Pearson correlation coefficient results 0.89 with *p*-value <0.0001) and the average duration of contacts (Pearson correlation coefficient results 0.56 with *p*-value <0.0001). This confirms, as already observed by Cattuto et al. [9], the association between number and intensity of connections suggesting that both these measures can be considered as representative of individual social behaviors defining super-connectors and, in turn, potential super-spreaders.

### Symptomatic episodes and influenza-like illness

Among the 74 considered individuals, 45 reported sore throat and cough, 18 fever, 56 running nose, congestion or sneezing, 28 nausea, vomiting or diarrhea. 15 individuals reported symptoms that can be classified as Influenza-like illness (ILI) according with definitions proposed by the World Health Organization (WHO), the European Centre for Disease Prevention and Control (ECDC), and the Centers for Disease Prevention and Control (CDC) [19–21]. The first ILI case was reported on January 13th, while the last one was recorded on March 4th. The number of consecutive days during which individuals reported an ILI ranges between 1 and 4 days resulting in an average duration of the symptomatic period of 1.73 days (sd 1.22).

### Human behavioral changes triggered by symptoms

**Fig. 3:**
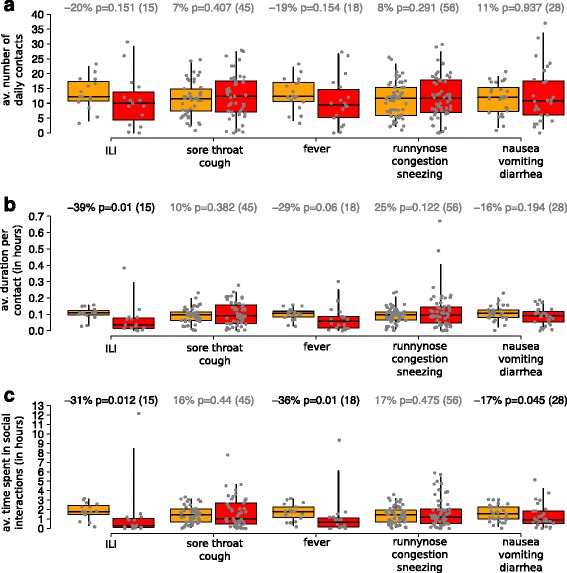
Distribution (2.5, 25, 50, 75, 97.5%) of **a** the average number of contacts per day of individuals who reported symptoms during days in which symptoms were reported (in *red*) and during days in which symptoms were not reported (*orange*); **b** as a) but for the average duration of contacts; **c** as a) but for the average daily time spent in social interaction. Percentage of change in social behavior triggered by different symptoms are shown on the top of each panel, along with the *p*-value corresponding to the performed paired Wilcoxon test and sample sizes associated with each symptomatic condition

Our analysis reveals that changes in the individual average number of daily contacts during days characterized by symptomatic conditions is not statistically significant for any of the symptom considered (see Fig. 3a). However, we found that Influenza-like illness may be responsible of a significant decrease in both the average duration of contacts and the daily time spent in social interactions (see Fig. 3b and c). Specifically, our results show that, during symptomatic conditions associated with Influenza-like illness, the duration of contacts is reduced on average by 39% (Wilcoxon test, *p*-value = 0.01), and the average daily time spent in social interactions is reduced by 31% on average (Wilcoxon test, *p*-value = 0.012). Our results suggest that this is mainly driven by the presence of fever, since individual activities significantly changes during days in which fever was recorded, while no remarkable differences can be detected neither when considering symptomatic events such as sore throat and cough, nor when considering runny nose, congestion and sneezing as potential triggers of a behavioral change. Specifically, our results suggest that the average daily time spent in social interactions is reduced on average by 36% as a consequence of fever only (Wilcoxon test, *p*-value < 0.01), along with a reduction of 29% in the mean duration of contacts (Wilcoxon test, *p*-value = 0.06). Finally, nausea, vomiting and diarrhea were also found to reduce the time spent in social interaction by 17% (*p*-value = 0.045), although such symptoms do not have a significant influence neither on the duration of each contact nor on the average number of daily contacts.

### Individual social interactions and potential risk factors for influenza

**Fig. 4:**
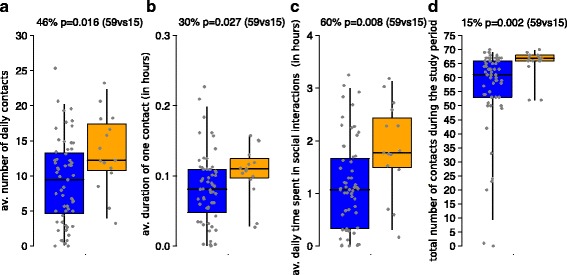
Distribution (2.5, 25, 50, 75, 97.5%) of **a** the average number of contacts per day recorded for individuals who reported symptoms associated with Influenza-like illness (in *orange*) and for individuals who did not (*blue*); **b** as a) but for the average duration of contacts; **c** as a) but for the daily time spent in social interactions; **d** as a) but for total number of contacts during the whole study period. Differences in social behavior associated with ILI cases are shown on the top of each panel, along with the *p*-value corresponding to the performed Wilcoxon test and the sample of the two mutually exclusive groups considered

In order to give some insights on how social behaviors can impact the risk of infection, we investigate differences in behavior between the study participants who reported an Influenza-like illness with respect to those who did not reported symptoms compliant with clinical influenza. As shown in Fig. 4, our results suggest that the former group of individuals spent on average 60% more time in social interactions (Wilcoxon test, *p*-value = 0.006), had 15% more connections with other individuals during the whole study period (Wilcoxon test, *p*-value = 0.002), had 46% larger number of contacts per day (Wilcoxon test, *p*-value = 0.016) lasting on average 30% longer (Wilcoxon test, *p*-value < 0.027).

### Epidemiological links associated with influenza-like illness

The investigation on possible influenza transmission events occurred among study participants shows that, among 15 individuals reporting symptoms compliant with clinical influenza, 7 could be classified as isolated symptomatic cases. More specifically, by considering potential transmission events caused by individuals’ physical proximity with observed clinical influenza cases, at least 7 separate chains of transmission were identified. This means that the time series of symptoms reported by study participants is not compliant with the hypothesis that a unique epidemic occurred within the network of study participants, exclusively sustained by symptomatic infections. Such inconsistency does not emerge when a fully connected network is considered. More details can be found in the Additional file 2: S2.

## Discussion

Our results show that symptoms associated with influenza can be responsible of a statistically significant reduction of both the average duration per contact (about 40% less) and the daily time spent in social interactions (about 30% less), possibly driven by the presence of fever. Changes in the daily number of contacts, triggered by symptoms associated with Influenza-like illness, were instead found not statistically significant. These change in the type of individuals’ interactions are particularly of interest given that the duration of contacts has been suggested to play a crucial role in shaping the transmission dynamics of infectious diseases [12, 14].

Our analysis reveals that a remarkable heterogeneity in terms of social behaviors can characterize individuals with similar age and daily routines. Interestingly, we found that individuals who experienced Influenza-like illness were characterized by a significantly higher social activity with respect to study participants who did not. In particular, a high number of person-to-person contacts and a large amount of time spent in proximity interactions can be considered as representative measures of human behavior defining super-connectors and super-spreaders and therefore suggested as potential risk factors for influenza infection.

Given that the onset of specific symptoms can reduce individuals’ social interactions, silent transmission though asymptomatic (and more active) individuals may contribute to spread the infection in the population. The presence of influenza viral shedding in patients with influenza who have very few or no symptoms has been recently confirmed [23]. Estimates on the prevalence of influenza asymptomatic carriers (i.e. infected individuals showing no symptoms) and influenza subclinical cases (i.e. symptomatic cases that do not meet the criteria for Influenza-like illness) ranges respectively from 5.2 to 35.5% and from 25.4 to 61.8% [24]. Nonetheless, it has been suggested that “silent spreaders” (i.e., individuals who are infectious while asymptomatic or pre-symptomatic) may be less important in the spread of influenza epidemics than previously thought [25].

The main limitation of this work relies on the small number of study participants considered, and on the highly specific network defined by sampling close proximity every six minutes. Indeed, in our analysis, social interactions of shorter duration and contacts occurred outside the student network were not accounted for. Relevant academic and extra-curricular activities might have not been covered by the study, either because the mobile phones could not be permanently on (e.g. during classes), or because of contacts with people not taking part to the study. As a consequence, any expectation that symptomatic cases of a given infection should be linked in a single chain of transmission may result inappropriate. In particular, the performed analysis on possible epidemiological links associated with Influenza-like illness recorded among study participants during the follow-up period suggests that less than 50% of observed cases can be ascribed to symptomatic infections occurred among study participants, and either asymptomatic cases or contacts with people not taking part to the study might have been important sources of infection. In particular, since no wash out of study participants was observed during the period under study, the spread of influenza among the considered individuals may have occurred either through infections generated outside the considered network of contacts or through transmission from asymptomatic infectious cases. This critical aspect does not emerge when a fully connected network is considered therefore strengthening the idea that, as suggested by Isella et al. [26] and Stehlé et al. [12], static aggregated networks are not suitable for the investigation of transmission chains within small groups of individuals, and that the assumption of homogeneous mixing within strongly connected communities could lead to erroneous epidemiological interpretations of the underlying transmission paths.

Although social connections recorded during the study period provide only partial information on individuals’ social behavior in the underlying community, the dormitory may still represent the preferential place where students live, cook and sleep [17] and it is likely that individuals who reported a significant higher social activity level with other study participants have been characterized by similar social behaviors in the general community.

## Conclusions

In this work we used data of 74 undergraduate students, collected in a dormitory of a North America campus during the 2009. The data include both physical proximity records obtained by Bluetooth sensors on individuals’ smartphones and self-reported surveys about the presence of specific symptoms. The carried out analysis shows that symptomatic conditions associated with Influenza-like illness can remarkably reduce the social activity of individuals. In particular, our findings suggest that changes in contact patterns caused by influenza are mainly driven by the onset of fever and mainly affect the duration of individuals’ contacts, rather than the number of individuals with whom they have social interactions. Obtained results highlight that, as already observed by Obadia et al. [12] and Smieszek et al. [27], the heterogeneity in social habits among individuals can significantly contribute in shaping differences among the individuals’ risk of infection. Interestingly, we found that, beyond the well-known role played by age in shaping individuals’ contact patterns [1, 11, 14], remarkable differences in social behavior can also emerge among individuals characterized by similar daily routines. Finally, our study suggests that transmission by asymptomatic cases can be crucial to sustain the spread of the infection in small groups and may contribute to a significant fraction of cases in the general population. The potential role played by silent influenza transmission claims for new studies aimed at investigating the probability of developing symptoms and the differential infectiousness between asymptomatic and symptomatic cases.

## Additional files

Additional file 1: S1. Data on proximity and symptoms records. (TXT 21495 kb)

Additional file 2: S2. Supplementary Text. (TXT 82 kb)

## Abbreviations

CDC: Centers for disease control and prevention
ECDC: European centre for disease control and prevention
H1N1: Influenza A (H1N1) virus
ILI: Influenza-like Illness
RFID: Radio-frequency Identification
UK: United Kingdom
WHO: World Health Organization
